# YKL-39 as a Potential New Target for Anti-Angiogenic Therapy in Cancer

**DOI:** 10.3389/fimmu.2019.02930

**Published:** 2020-01-22

**Authors:** Julia Kzhyshkowska, Irina Larionova, Tengfei Liu

**Affiliations:** ^1^Medical Faculty Mannheim, Institute of Transfusion Medicine and Immunology, University of Heidelberg, Mannheim, Germany; ^2^German Red Cross Blood Service Baden-Württemberg—Hessen, Mannheim, Germany; ^3^Laboratory of Translational Cellular and Molecular Biomedicine, National Research Tomsk State University, Tomsk, Russia; ^4^Cancer Research Institute, Tomsk National Research Medical Center of the Russian Academy of Sciences, Tomsk, Russia

**Keywords:** YKL-39, chitinase-like proteins, cancer, angiogenesis, chemotactic activity, tumor-associated macrophages, neoadjuvant chemotherapy

## Abstract

YKL-39 belongs to the evolutionarily conserved family of Glyco_18-containing proteins composed of chitinases and chitinase-like proteins. Chitinase-like proteins (CLPs) are secreted lectins that lack hydrolytic activity due to the amino acid substitutions in their catalytic domain and combine the functions of cytokines and growth factors. One of the major cellular sources that produce CLPs in various pathologies, including cancer, are macrophages. Monocytes recruited to the tumor site and programmed by tumor cells differentiate into tumor-associated macrophages (TAMs), which are the primary source of pro-angiogenic factors. Tumor angiogenesis is a crucial process for supplying rapidly growing tumors with essential nutrients and oxygen. We recently determined that YKL-39 is produced by tumor-associated macrophages in breast cancer. YKL-39 acts as a strong chemotactic factor for monocytes and stimulates angiogenesis. Chemotherapy is a common strategy to reduce tumor size and aggressiveness before surgical intervention, but chemoresistance, resulting in the relapse of tumors, is a common clinical problem that is critical for survival in cancer patients. Accumulating evidence indicates that TAMs are essential regulators of chemoresistance. We have recently found that elevated levels of YKL-39 expression are indicative of the efficiency of the metastatic process in patients who undergo neoadjuvant chemotherapy. We suggest YKL-39 as a new target for anti-angiogenic therapy that can be combined with neoadjuvant chemotherapy to reduce chemoresistance and inhibit metastasis in breast cancer patients.

## Introduction

YKL-39 belongs to the family of Glyco_18-containing proteins composed of chitinases and chitinase-like proteins. Chitinases comprise the Glycosyl hydrolase (GH) 18 family; their name originates from their ability to cleave chitin polymers into oligosaccharides of different sizes and release monosaccharides from the end of chitin polymer (1, 2). Chitin is the second most abundant polysaccharide in nature (after cellulose) and is found in the cell walls of fungi and the exoskeletons of crustaceans and insects (3–5). The chitinases are produced by the lower life forms as a defense mechanism against infection with chitin-containing organisms (6, 7). Mammals cannot synthesize chitin, but several chitinases and chitinase-like proteins have been identified in rodents and in humans. In humans, two functional chitinases—Acidic Mammalian Chitinase (AMCase) and Chitotriosidase (CHIT1)—have been found. AMCase is induced by IL-13 and is found in allergic inflammations such as asthma (8, 9). Chitotriosidase is expressed by phagocytic cells and is a biomarker for Gaucher's disease, a lysosomal storage disease that involves the dysfunctional metabolism of sphingolipids (10, 11).

Chitinase-like proteins (CLPs), as well as chitinases, possess Glyco_18 domains, but they lack enzymatic activity (12). In mammals, the following CLPs have been identified: YKL-40 (13), YKL-39 (14), SI-CLP (15), YM1, and YM2 (16). Of these, YKL-39 is present only in humans but absent in rodents, while YM1 and YM2 are only present in rodents (12).

**Figure 1:**
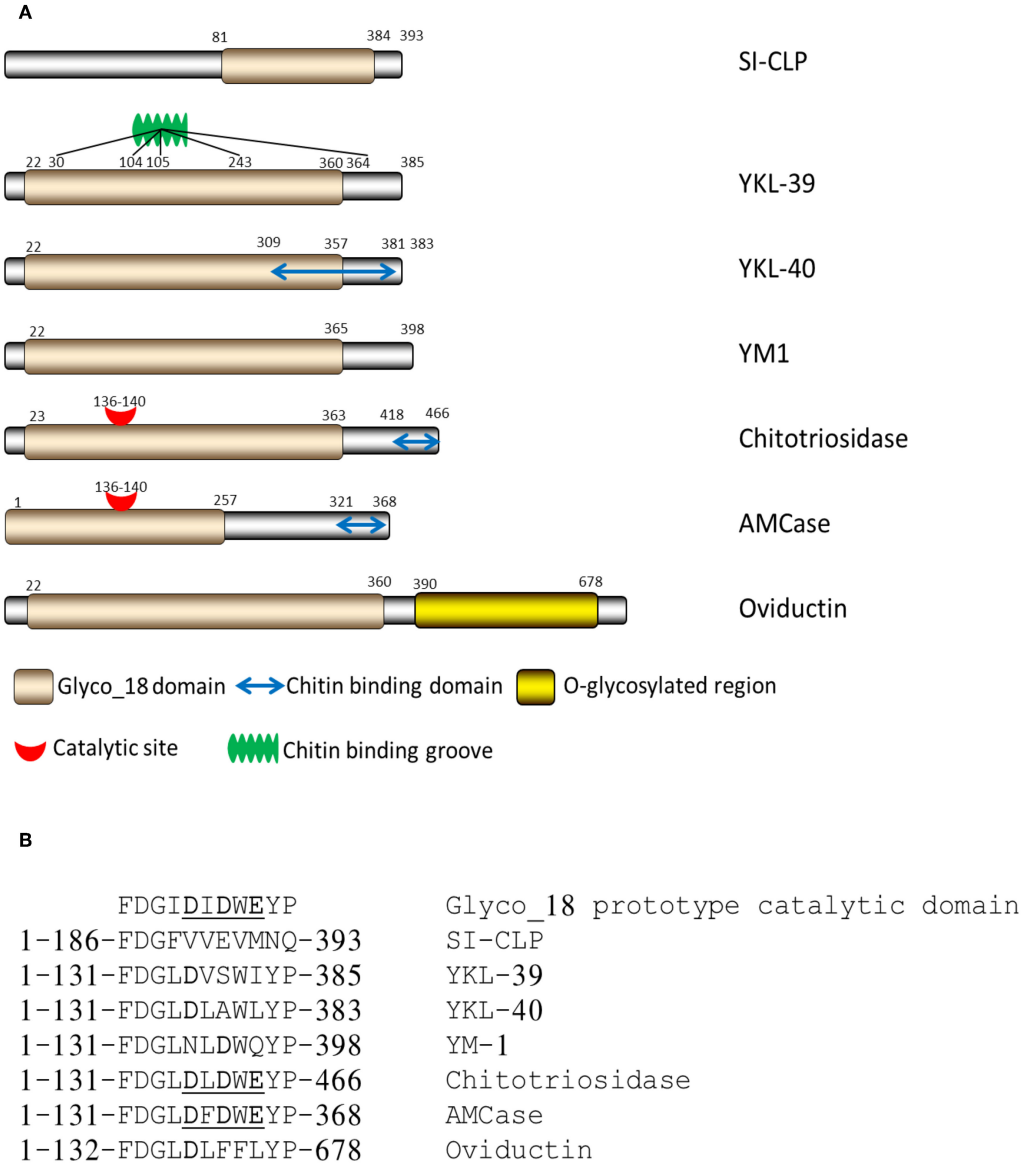
Structure of mammalian chitinases and chitinase-like proteins. **(A)** Domain organization of Glyco_18-containing proteins. **(B)** Critical amino acids in catalytic sites of mammalian Glyco_18-containing proteins (12), Copyright by de Gruyter.

CLPs lack enzymatic activity due to the substitution of the critical catalytic residue (glutamic acid) at the end of the DxxDxDxE conserved motif with either leucine, isoleucine, or tryptophan (Figure 1) (12).

The sugar-binding properties of CLPs are attributed to the Glyco_18 domain of CLPs (Table 1). Lectin properties define the interactions of CLPs with glycoproteins on the cell surface and with specific carbohydrate molecules in the extracellular matrix. For YKL-40, lectin properties have been identified to be critical for its interaction with syndecan-1 and αvβ3 integrin, resulting in the activation of the ERK1/2 pathway and vascular endothelial growth factor (VEGF) production in endothelial cells (25, 26). Moreover, SI-CLP was shown to bind lipopolysaccharide (LPS) *in vitro* and thereby to neutralize the toxic effect of LPS on macrophages (23). By applying a glycan microarray, performed at the Consortium of Functional Glycomics, the chitooligosaccharides were identified as the best ligands of YKL-39 (17). Structural analysis demonstrates that YKL-39 interacts with chitooligosaccharides through hydrogen bonds and hydrophobic interactions, and compared with other GH-18 members, YKL-39 has the least extended chitin-binding cleft (18). However, the biological relevance of these interactions is questionable, since chitin is not synthesized by mammals, and the tissue expression of YKL-39 rather precludes contact with chitooligosaccharides as a component of the nutrition or pathogens (17).

**Table 1:** Lectin properties of CLPs.

**CLP**	**Carbohydrate-binding**	**Method of analysis**	**References**
YKL-39	Chitooligosaccharides, (GlcNac)5, and (GlcNac)6	Glycan array screen and intrinsic tryptophan fluorescence	(17)
	Chitooligosaccharides	Isothermal titration calorimetry (ITC)	(18)
YKL-40	Type I collagen	Affinity chromatography and surface plasmon resonance	(19)
	Chitooligosaccharides	Protein X-ray crystallography	(20)
	(GlcNac)5 and (GlcNac)4	Western blotting	(21)
	Heparin	Heparin affinity and HPLC chromatograph	(22)
SI-CLP	Galactosamine, glucosamine, chitooligosacharide, (GlcNac)4, ribose, and mannose	Isothermal titration calorimetry (ITC)	(23)
YM1	Glucosamine, galactosamine, and glucosamine polymers	Surface plasmon resonance	(24)

## YKL-39 Identification and Expression in Pathology

YKL-39 was first identified when found to be produced in high amounts by synoviocytes and chondrocytes (14, 27) and was suggested as a circulating biomarker for osteoarthritis (OA) (14, 27, 28). Increased YKL-39 mRNA levels were also detected in the microglia of Alzheimer patients (29). The detection of YKL-39 in cerebrospinal fluid was suggested to be a potential prognostic biomarker in the early stage of multiple sclerosis (30, 31). Also, YKL-39 mRNA levels were significantly increased in the hippocampus in simian immunodeficiency virus encephalitis (SIVE) and HIV encephalitis (HIVE) (32). These data suggested the role of YKL-39 in both neurodegeneration and chronic inflammatory diseases of the brain. We have recently identified that YKL-39 is expressed in human breast cancer (33), and these data are discussed in the context of the role of CLPs in tumor progression and response to therapy in the following paragraphs.

## Biological Activities

Biological activities of chitinase-like proteins related to tumor progression include chemotactic activity, growth factor activity, induction of cytokine secretion, and stimulation of angiogenesis. YKL-39 was identified to combine monocyte chemotactic and pro-angiogenic activities (33), and these biological activities will be discussed in our review.

## Chemotactic Activity

Several chitinase-like proteins were demonstrated to have chemotactic activities. YM1 was first identified as eosinophil chemotactic protein (ECF-L) (34). YM1 attracted T lymphocytes and bone marrow polymorphonuclear leukocytes *in vitro* and induced selective extravasation of eosinophils in a mouse model (34). Microglia-secreted YM1 was suggested to be involved in eosinophilic meningitis and meningoencephalitis caused by Angiostrongylus cantonensis infection (35). YM1 and YM2 were strongly induced in a mouse model for proliferative dermatitis characterized by the accumulation of eosinophils in the skin (36).

Human YKL-40 was reported to have chemotactic activity toward different cell types. Nishikawa et al. showed that YKL-40 is associated with vascular smooth muscle cell (VSMC) migration and invasion into the gelatinous matrix (22). YKL-40 expressed in human colon cancer SW480 cells enhanced the migration of human monocyte-like THP-1 cells and human umbilical vein endothelial cells (HUVEC). The expression of YKL-40 was associated with macrophage infiltration and micro-vessel density (MVD) in the tumors of human colorectal cancer patients and in a xenograft mouse model (37). YKL-40 was also found to contribute to the migration of bronchial smooth muscle cells indirectly by inducing the expression of IL-8 (38).

**Figure 2:**
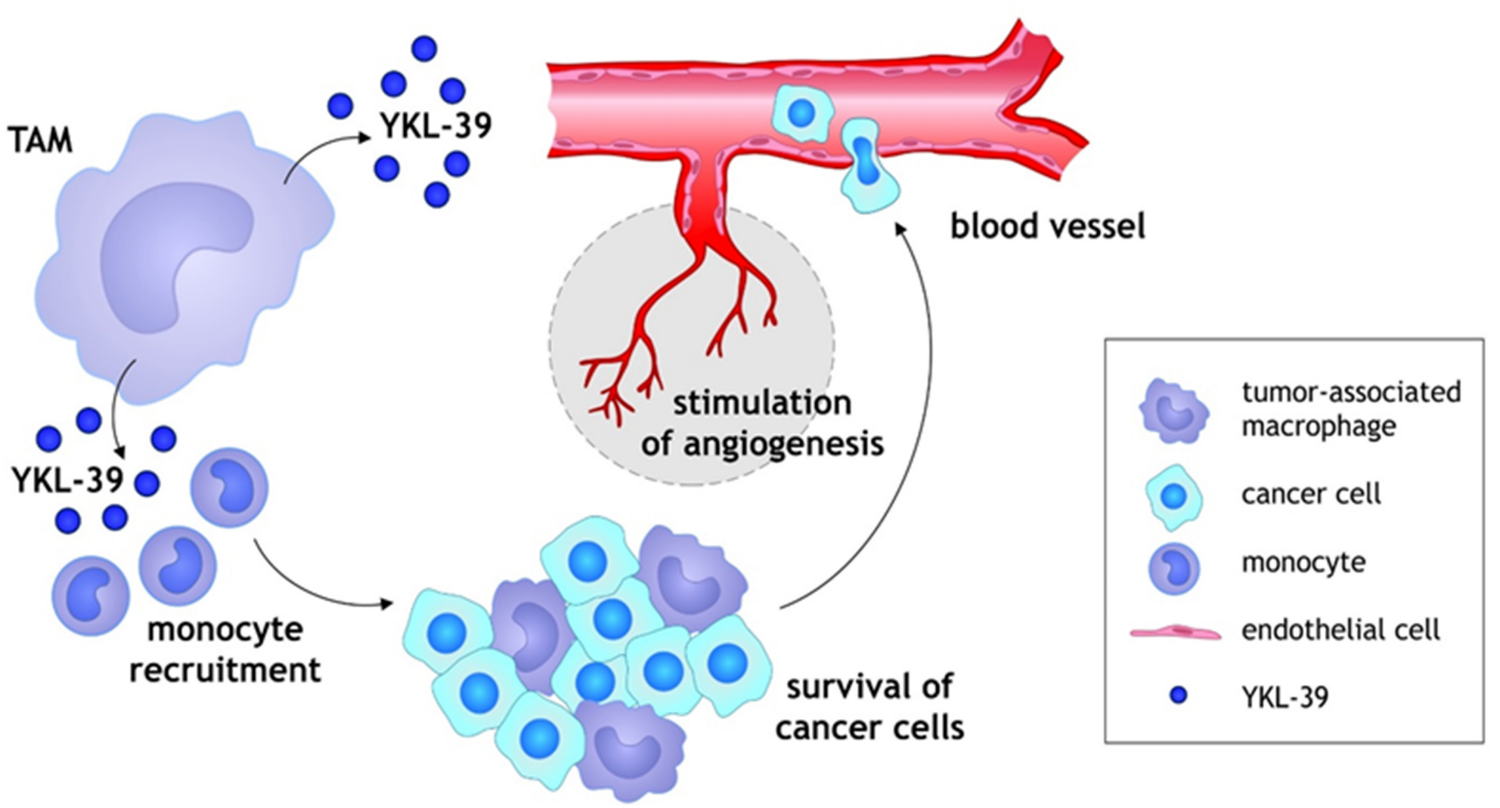
Schematic illustration of YKL-39 activity in cancer. Monocytes are recruited into growing tumors by chemotactic factor YKL-39 secreted by TAMs in the tumor microenvironment, where TAMs support the survival and growth of cancer cells. Monocytes differentiate in the tumor tissue into TAMs, which are key inducers of the angiogenic switch. YKL-39 possesses pro-angiogenic activity and causes stimulation of angiogenesis that can lead to the intensive intravasation of cancer cells to the blood vessels.

We have recently demonstrated that purified YKL-39 strongly induces the migration of freshly isolated human CD14+ monocytes (Figure 2) (33). YKL-39 was active at the concentration of 100 ng/ml corresponding to the biologically active concentration of YKL-40, 90.3 ± 8.2 ng/ml, in patients with OA (39). After 3 h of migration, the effect of YKL-39 was comparable to the effect of the major monocyte chemotactic factor CCL2 if used at the same concentration. Monocytes are intensively recruited into growing tumors by chemotactic factors secreted by tumor cells and stromal cells in the tumor microenvironment, where both tumor-associated macrophages (TAMs) and cancer cells serve as sources of chemotactic factors such as CCL2 (40, 41). Monocytes differentiate in the tumor tissue into tumor-associated macrophages, which are key inducers of the angiogenic switch (42). The strong chemotactic activity of YKL-39 makes it an attractive candidate to consider as a target to reduce monocyte recruitment into the tumor tissue.

## Angiogenesis

YKL-39 was identified by us as a strong pro-angiogenic factor *in vitro*. Tumor angiogenesis is a crucial process for supplying rapidly growing tumors with essential nutrients and oxygen (41). Monocytes recruited to the tumor site and programmed by tumor cells are known as TAMs, which are the primary source of pro-angiogenic factors (41, 43). TAMs produce a variety of pro-angiogenic factors under the hypoxic condition in tumor sites, for example, VEGF, which promotes migration of endothelial cells and macrophages toward tumor areas (40, 44).

VEGF is the prototypical proangiogenic factor that induces vascular permeability and increased migration and proliferation of endothelial cells, making it a major target for therapy (45). One of the main anti-angiogenic approaches is to block VEGF using a monoclonal antibody (bevacizumab). Other drugs include VEGF pathway inhibitors such as small-molecule tyrosine kinase inhibitors (sunitinib, sorafenib, pazopanib, regorafenib, lenvatinib, and vandetanib), a soluble VEGF decoy receptor (aflibercept), a human monoclonal antibody against VEGFR-2 (ramucirumab), and others (45, 46). Administering bevacizumab in combination with chemotherapeutic agents showed improved survival of patients with colorectal cancer, ovarian cancer, and lung cancer in comparison with chemotherapy alone (46–48). However, there is resistance against anti-VEGF medication, which includes several mechanisms, such as the activation and upregulation of alternative proangiogenic pathways, the recruitment of bone marrow-derived proangiogenic cells, and the adoption of alternative angiogenic mechanisms (45). Several studies have demonstrated TAM accumulation in the tumor mass after chemotherapy and antiangiogenic therapy. Thus, Dalton et al. showed that recruitment of macrophages to the TME after anti-VEGF treatment leads to tumor growth as a mechanism of resistance to therapy but that depletion of macrophages inhibited tumor growth and improved the survival of tumor-bearing mice (49). The vascular disrupting agent combretastatin A4-P causes the increased production of CSF-1, CCL2, and CXCL12 that increases monocyte recruitment and TAM accumulation in tumor sites (50). In mouse mammary tumors, chemotherapy increased the expression of CSF-1 by tumor cells, followed by the recruitment of macrophages (51). Thus, chemotherapy and anti-VEGF therapy have disadvantages such as TAM accumulation and treatment resistance, and additional new therapeutic approaches need to be developed.

Chitinase-like protein YKL-40 has already been shown to be involved in tumor angiogenesis in several studies. It was reported that gp38k (porcine homolog protein of YKL-40) promotes the migration and spreading of VSMCs *in vitro* (22). The expression of YKL-40 in MDA-MB-231 breast cancer cells and HCT-116 colon cancer cells is also associated with tube formation in an extensive angiogenic phenotype mouse model (26). Recombinant YKL-40 protein was also found to induce angiogenesis of vascular endothelial cells *in vitro* (52). A correlation between blood vessel density and YKL-40 expression has also been observed in human breast cancer patients (53). The YKL-40-induced pro-angiogenic effect was VEGF-independent, suggesting that YKL-40 and VEGF individually promote endothelial cell angiogenesis (26). However, a long-term blockade of VEGF may result in angiogenic compensative tumor cell activities by inducing YKL-40 (54). It is most likely that blockade of one angiogenic factor induces the expression of other potent angiogenic factors to maintain tumor vascularization.

YKL-39 has a high structural similarity to YKL-40. Therefore, we considered that YKL-39 can act as a pro-angiogenic factor in cancer. We have performed tube formation assay using HUVEC cells and found that YKL-39 exerts a strong pro-angiogenenic effect through direct activation of vascular endothelial cells (Figure 2). Recombinant YKL-39 at a concentration of 100 ng/ml significantly induced tube formation in HUVEC cells *in vitro* (33). This data indicated that YKL-39 can directly induce angiogenesis and that YKL-39-expressing TAMs can serve as a source of angiogenic factors in the tumor microenvironment.

## Macrophages are a Major Source of Chitinase-Like Proteins

Pathological programming of macrophages is crucial for the development of major types of life-threatening disorders, including cancer and cardiovascular and neurodegenerative disorders (55–59). Macrophages regulate intratumoral immune responses and the progression of atherosclerosis by the secretion of cytokines, growth factors, enzymes, and extracellular matrix proteins (41, 55, 60). Two major directions of macrophage polarization are known: pro-inflammatory M1 macrophages with antitumor properties and anti-inflammatory M2 macrophages with pro-tumor functions. In most solid tumors, macrophages are represented by the M2 phenotype, which supports tumor growth, angiogenesis, and metastatic spread (61, 62). Macrophages serve as a major source of all murine and human chitinase-like proteins (Table 2). However, the expression of YKL-40 is not restricted to macrophages (TAMs) and has been found in human small cell lung cancer (63), microglia from Alzheimer's disease patients (29), and other cell types. The expression of SI-CLP was detected in peripheral blood mononuclear cells from rheumatoid arthritis (RA) patients (68).

**Table 2:** Expression of chitinase-like proteins in macrophages.

**CLP**	**Macrophage type**	**Method of analysis**	**References**
YKL-40	Human primary monocyte-derived macrophages stimulated by IFN-γ	RT-PCR	(15)
	Microglia in Alzheimer's disease patients	RT-PCR	(29)
	Human peritumoral macrophages and murine lung macrophages	RT-PCR	(63)
	Human macrophages in pulmonary sarcoid granulomas	Immunohistochemical staining	(64)
	Peritumoral macrophages in human small cell lung cancer	Immunohistochemical staining	(63)
	Human primary monocyte-derived macrophages stimulated by GM-CSF or M-CSF	RT-PCR/ELISA/ Immunofluorescence staining	(65)
	Murine pulmonary macrophages	RT-PCR/ELISA	(66)
	Human M1 polarized macrophages stimulated by LPS and IFN-γ	RT-PCR	(67)
SI-CLP	Human primary monocyte-derived macrophages stimulated by IL-4+dexamethasone	RT-PCR/Western blotting/Immunofluorescence staining	(15)
	Murine bone marrow-derived macrophages	RT-PCR/Western blotting	(68)
	PMA-treated THP-1 macrophages	RT-PCR/Western blotting	(68)
	THP-1, Mono-Mac-6 cells	RT-PCR/Western blotting	(15)
YKL-39	Human primary monocyte-derived macrophages stimulated by TGF-beta and IL-4	RT-PCR	(33, 69)
	Alternatively activated microglia in Alzheimer's disease patients	RT-PCR	(29)

Elevated levels of YKL-39 gene expression were detected in microglia of Alzheimer's patients (29). In *in vitro* experimental models, expression of CLPs depends on the activation state of macrophages (M1 or M2). YKL-40 expression is elevated during the differentiation process of human macrophages, and macrophage differentiation factors GM-CSF or M-CSF have been shown to induce YKL-40 expression (65, 70). It was identified that in human monocyte-derived macrophages, IFN-γ and LPS are strong inducers of YKL-40 gene expression (15, 67). In contrast, expression of SI-CLP is induced by IL-4 and dexamethasone on both the mRNA and protein levels in human monocyte-derived macrophages (15). YKL-39 expression is strongly induced by TGF-beta, an essential regulatory cytokine of the tumor microenvironment (33).

The secretion of SI-CLP and YKL-39 at least partially depends on their transport into the secretory lysosomes, mediated by their intracellular sorting by stabilin-1 (15, 33). Lysosomes are organelles with complex functions involved in the cell death, immunity, signaling, and stress responses (71–73) that not only participate in digesting extracellular material internalized by endocytosis and intracellular components sequestered by autophagy but also secrete their contents by fusing with the plasma membrane (72). Two types of lysosome-contained proteins are necessary for their functions: soluble hydrolases and integral lysosomal membrane proteins. More than 60 hydrolases have been identified and characterized, some of which play an important role in tumor progression (72, 74). The best investigated lysosomal hydrolases are the cathepsin proteases, which are subdivided into three groups based on the active site of the amino acids and the catalytic activity: serine cathepsins (cathepsins A and G), cysteine cathepsins (cathepsins B, C, F, and H), and aspartic cathepsins (cathepsins D and E) (72). It has been suggested that cathepsins could either promote or suppress tumor growth; the cytosolic cathepsins inhibit tumor growth by activating the apoptotic pathway (75), whereas, in contrast, the extracellular cathepsins promote tumor growth through degradation of basement membrane and activation of other pro-tumorigenic proteins (76). Cathepsins B and E have been proved to be involved in cancer progression and metastasis in different types of cancer, such as breast cancer and pancreatic cancer (69, 77). Glyco_18 domain-containing proteins were also found by us and others to be sorted via the endosomal/lysosomal system and secreted by activated macrophages (4, 15, 78). Chitotriosidase was seen to be comparable to cathepsin D in lysosomal vesicles in macrophages (78). We identified that LAMP+CD63+lysosomes are major sites of SI-CLP localization in human IL4 and dexamethasone-stimulated M2 macrophages (15). Sorting of newly synthesized SI-CLP from the biosynthetic to the lysosomal pathway was mediated by stabilin-1. Similarly, we found that YKL-39 is sorted into LAMP-1 positive and secretion-committed CD63 positive lysosomes in human IL-4+TGF-beta-stimulated macrophages (33). The mechanistic role of stabilin-1 in the intracellular sorting of YKL-39 was confirmed using the HEK293-YKL-39-FLAG cell line, where YKL-39 is miss-sorted into the globular structures and localized in the nuclear area. Transient overexpression of recombinant stabilin-1 in this model cell line resulted in the re-distribution of YKL-39 into the cytoplasm, and this effect was similar to our previously published data demonstrating the role of stabilin-1 in the intracellular sorting of SI-CLP in a H1299 cell model (15). Protein–protein interaction studies demonstrated that the extracellular fasciclin domain 7 domain of stabilin-1 directly interacts with SI-CLP and YKL-39 (15, 33). Therefore, YKL-39, similarly to SI-CLP, can be targeted by stabilin-1 into the lysosomal secretory pathway in human alternatively activated macrophages.

YKL-39 was identified by us to be produced by human macrophages *in vitro* and in the tumor microenvironment (33, 79). *In vitro*, TGFbeta was a key inducer of YKL-39 gene expression, and release in primary macrophages propagated up to 24 days (33). During tumor growth and progression, a significant amount of TGF-beta is produced by cancer and stromal cells and secreted into the tumor microenvironment (80). Increased expression of TGF-beta was shown to correlate with the malignancy of different cancers (81, 82). Therefore, TGF-beta is considered to play a major role in the initiation and progression of cancer by affecting the proliferation, apoptosis, and differentiation of cancer cells in the tumor microenvironment (83). Using a monoclonal-aYKL-39 antibody generated by us, we have identified that in human breast cancer, YKL-39 is expressed on TAMs, but, in contrast to YKL-40, not on cancer cells.

## Chitinase-Like Proteins in Cancer

Accumulating data reveals that CLPs play a role in the progression of different types of cancer. Elevated levels of circulating YKL-40 are related to poor outcome or short disease-free survival in glioblastoma, melanoma, ovarian, breast, colon, lung, and prostate cancers in humans (52, 84–92). Moreover, in breast cancer, elevated serum levels of YKL-40 have been used as a prognostic biomarker (84). The adhesive and invasive abilities of U87MG glioblastoma cells were significantly inhibited when endogenous expression of YKL-40 was blocked (93). YKL-40 was also induced during pulmonary melanoma metastasis, and this induction was mediated by Sema7a (90, 94). Overexpression of YKL-40 and YM1/2 was observed in the pre-neoplastic phase of a latent membrane protein 1 (LMP1) viral oncogene-expressing transgenic mouse model, which is associated with carcinogenic progression (95). In breast cancer, YKL-40 may support cancer progression and facilitate angiogenesis, as experimental knock-down of YKL-40 in tumorigenic breast epithelial cell line D492HER2 resulted in reduced migration and invasion as well as reduced ability to induce angiogenesis *in vitro* (96). Targeting of YKL-40 as a potential therapeutic approach has been evaluated in melanoma and glioblastoma mouse models. Application of anti-YKL-40 antibody in the U87 glioblastoma mouse models resulted in the suppression of xenograft tumor growth as well as angiogenesis (97). However, an opposite result was seen in BALB/c-scid mice injected with human melanoma cells; the tumor growth was enhanced after anti-YKL-40 antibody treatment (98). The contradictory results between glioblastoma and melanoma mouse models can be explained by the different mouse strains and antibodies used in the studies.

SI-CLP was shown to induce the secretion of IL-1β, IL-6, IL-12, and IL-13 in PMA-treated THP-1 cells, suggesting that it may serve as a regulator of inflammation and in the tumor microenvironment (68). The regulatory effect of SI-CLP is not clear yet since the cytokines induced by SI-CLP can either promote (IL-1β, IL-6, IL-13) or suppress (IL-12) tumor progression (99, 100).

Information about the potential role of YKL-39 in cancer is still very limited. Serial Analysis of Gene Expression (SAGE) revealed that YKL-39 expression is elevated in the II–IV grades of glial tumors (101). The expression of YKL-39 was detected in the majority of glioblastomas (19 of 27 samples analyzed) by Northern blot analysis and demonstrated on the protein level by Western blotting (102). Our recent study has demonstrated that YKL-39 is expressed in human breast cancer, and its expression level were indicative of metastatic spread in patients who underwent neoadjuvant chemotherapy, as discussed below.

## Chemotherapy, TAMs and Chitinase-Like Proteins

Chemotherapy (CT) is a common strategy for reducing tumor size and aggressiveness before surgical intervention (103). However, only a subset of patients respond efficiently to neoajuvant chemotherapy (NAC). Chemoresistance and chemotherapy-induced immunosuppression can result in the relapse of tumors and are critical for survival in cancer patients (49, 104). Numerous studies have examined the molecular mechanisms that promote the chemoresistance of cancer cells, such as the induction of anti-apoptotic regulators, ABC transporters, aberrant transcription factor nuclear factor-κB (NF-κB) activity, and the mechanisms of damaged DNA repair (104–106). Evidence is accumulating that the tumor microenvironment, and TAMs in particular, is critical to the response to chemotherapy (107, 108). TAMs may contribute to resistance to therapy and facilitate tumor progression via macrophage-induced suppression of T cell immunity, maintenance of tumor cell survival, and stimulation of tumor revascularization (50, 108, 109). Chemotherapeutic agents can edit macrophages in tumor-protective or antitumor directions, where three major mechanisms must be considered: (1) changes in the macrophage phenotype; (2) induced recruitment of monocytes or macrophages to the tumor site; and (3) systemic depletion of monocytes/macrophages (110). Numerous data demonstrate that chemotherapy interacts with macrophages; however, the mechanisms of the direct action of chemotherapeutic drugs on TAMs, as well as the mechanisms of TAM-mediated chemoresistance in tumors, still require in-depth investigation. The profile of immune cell subpopulations in the tumor microenvironment can help to identify a group of patients that are more sensitive/or resistant to neoadjuvant chemotherapy to improve treatment regimens.

Several studies have identified correlations between levels of CLPs and the efficiency of chemotherapy. For example, a high plasma level of YKL-40 was associated with shorter progression-free survival (PFS) and shorter overall survival (OS) in 140 patients with chemotherapy-resistant ovarian cancer treated with bevacizumab (111). A similar result was obtained in prostate cancer, where high serum YKL-40 levels were associated with shorter OS and disease-specific survival (DSS) in 109 patients who received first-line treatment with docetaxel (DOC). Moreover, YKL-40 serum levels were significantly higher in DOC-resistant patients (112). In another study, 120 patients with small cell lung cancer with high levels of serum YKL-40 had a shorter PFS and OS than those with low levels of serum YKL-40 (113). YKL-40 levels were significantly decreased after chemotherapy (cisplatin with etoposide or cisplatin with irinotecan). However, patients with high serum YKL-40 showed a poorer response to chemotherapy than those patients with low serum YKL-40. Of the 81 patients with high serum YKL-40, only 46% responded to chemotherapy with either complete response or partial remission. Of 39 patients who had low serum YKL-40, 70% exhibited a response to chemotherapy (*p* = 0.031) (113). The question of the exact role of CLPs in the response of cancer cells, macrophages, and the intratumoral vasculature to chemotherapeutic agents remains open.

A recent study of 195 patients in the European cohort with pancreatic ductal adenocarcinoma indicated single-nucleotide polymorphisms (SNP) in YKL39 that was associated with tumor-associated survival after pancreatic resection. Individuals who were homozygous for the minor A allele of SNP rs684559 (YKL-39) had an increased risk for tumor-associated death compared with patients with at least 1 G allele of rs684559 (protective phenotype) (114). Our recent study for the first time demonstrated the prognostic role of YKL-39 in cancer metastasis in breast cancer patients after neoadjuvant therapy (33). In patients with metastases, the expression levels of YKL-39 in tumor tissue obtained after NAC were more than 6 times higher than in the patients without metastases. Significantly higher expression levels of YKL-39 were found in patients with stable disease or progressive disease than in patients with the objective response (partial response). Our data demonstrated that elevated levels of YKL-39 in tumor tissues after NAC are indicative of poor prognosis.

Taking into consideration that YKL-39 was demonstrated by us as a pro-angiogenic factor and chemoattractant for monocytes, we suggest that YKL-39 is a promising target for cancer therapy and that targeting of YKL-39 can be considered in combination with NAC in breast cancer patients in order to reduce the risk of metastasis formation.

## Author Contributions

JK has structured the article, analyzed the literature, and wrote the manuscript. IL has analyzed the literature and wrote the manuscript. TL has analyzed the literature and wrote the manuscript.

### Conflict of Interest

The authors declare that the research was conducted in the absence of any commercial or financial relationships that could be construed as a potential conflict of interest.
